# Multi-transcriptomics analysis of microvascular invasion-related malignant cells and development of a machine learning-based prognostic model in hepatocellular carcinoma

**DOI:** 10.3389/fimmu.2024.1436131

**Published:** 2024-08-08

**Authors:** Haoran Huang, Feifeng Wu, Yang Yu, Borui Xu, Dehua Chen, Yuwei Huo, Shaoqiang Li

**Affiliations:** Center of Hepato-Pancreato-Biliary Surgery, The First Affiliated Hospital of Sun Yat-sen University, Guangzhou, Guangdong, China

**Keywords:** hepatocellular carcinoma, microvascular invasion, prognostic model, tumor microenvironment, scRNA-seq, spatial transcriptome

## Abstract

**Background:**

Microvascular invasion (MVI) stands as a pivotal pathological hallmark of hepatocellular carcinoma (HCC), closely linked to unfavorable prognosis, early recurrence, and metastatic progression. However, the precise mechanistic underpinnings governing its onset and advancement remain elusive.

**Methods:**

In this research, we downloaded bulk RNA-seq data from the TCGA and HCCDB repositories, single-cell RNA-seq data from the GEO database, and spatial transcriptomics data from the CNCB database. Leveraging the Scissor algorithm, we delineated prognosis-related cell subpopulations and discerned a distinct MVI-related malignant cell subtype. A comprehensive exploration of these malignant cell subpopulations was undertaken through pseudotime analysis and cell-cell communication scrutiny. Furthermore, we engineered a prognostic model grounded in MVI-related genes, employing 101 algorithm combinations integrated by 10 machine-learning algorithms on the TCGA training set. Rigorous evaluation ensued on internal testing sets and external validation sets, employing C-index, calibration curves, and decision curve analysis (DCA).

**Results:**

Pseudotime analysis indicated that malignant cells, showing a positive correlation with MVI, were primarily concentrated in the early to middle stages of differentiation, correlating with an unfavorable prognosis. Importantly, these cells showed significant enrichment in the MYC pathway and were involved in extensive interactions with diverse cell types via the MIF signaling pathway. The association of malignant cells with the MVI phenotype was corroborated through validation in spatial transcriptomics data. The prognostic model we devised demonstrated exceptional sensitivity and specificity, surpassing the performance of most previously published models. Calibration curves and DCA underscored the clinical utility of this model.

**Conclusions:**

Through integrated multi-transcriptomics analysis, we delineated MVI-related malignant cells and elucidated their biological functions. This study provided novel insights for managing HCC, with the constructed prognostic model offering valuable support for clinical decision-making.

## 1 Introduction

Hepatocellular carcinoma (HCC) accounts for over 80% of liver cancer cases and is the sixth most commonly diagnosed cancer globally. Despite the continuous advancement of diagnostic and therapeutic strategies for HCC, including systemic treatments such as targeted therapy and immune checkpoint inhibitor therapy, liver cancer still ranks third in terms of cancer-related mortality. This is largely attributed to its high rates of metastasis and recurrence (1–3). Within 5 years following hepatectomy, HCC patients experience a recurrence rate exceeding 50%, and the prognosis for recurrent patients is significantly worse than for those without recurrence (4, 5).

Microvascular invasion (MVI) is characterized by the infiltration of malignant cells into the microvasculature surrounding the liver tumor. As a critical risk factor for tumor recurrence and unfavorable prognosis in HCC patients, MVI is commonly observed in HCC cases (6). It’s important to note that HCC patients with MVI exhibit significantly lower disease-free survival (DFS; date of randomization or treatment to date of first recurrence or death) and overall survival (OS; date of randomization or treatment to date of death as a result of any cause) rates compared to those without MVI (7–11). Therefore, comprehensive research into the mechanisms underlying MVI occurrence is essential for guiding clinical treatment and management. Previous studies have suggested that the invasion and metastasis of HCC are closely associated with the tumor microenvironment (TME) (12). The TME of HCC, which encompasses various cell types such as stromal cells, immune cells, and malignant cells, is highly diverse. Notably, within the TME, there are small yet functionally unique cell subpopulations, including stem cell-related malignant cells (13). This raises the question of whether the occurrence and progression of MVI are also influenced by similar tumor subpopulations.

Technological advancements have provided insights into the gene expression profiles of tumor tissues through bulk transcriptome sequencing, while single-cell transcriptome sequencing has offered a more precise understanding of the roles played by different cells. Recently, spatial transcriptomics has offered spatial information that single-cell transcriptomics alone cannot provide (14). The combined application of these sequencing data will facilitate an in-depth study of the TME (15). In this study, we conducted a thorough analysis of the potential mechanisms underlying the occurrence and development of MVI in HCC by utilizing bulk transcriptome sequencing and clinical data from TCGA and HCCDB databases, in conjunction with single-cell transcriptome data provided by Li et al. (16) and Lu et al. (17), as well as spatial transcriptome data provided by Wu et al. (18).

With the advancement of genomics, constructing patient prognostic models based on gene expression profiles has proven effective (19). Compared to traditional modeling, machine learning algorithms have shown better fitting effects on the same data and are gradually widely applied in the biomedical field (20, 21). The combination of different algorithms falls under stacking algorithms, demonstrating excellent predictive performance. Drawing inspiration from Liu et al.’s publication, which integrated 10 machine learning algorithms, we used 101 algorithm combinations to determine the optimal modeling (22). We applied the leave-one-out cross-validation (LOOCV) framework to avoid overfitting. The final prognostic model based on MVI-related genes (MVIRGs) demonstrated strong predictive performance and clinical decision-making significance.

## 2 Materials and methods

### 2.1 Data sources used for analysis

The bulk RNA-seq dataset comprising 374 tumor tissue samples and 50 normal tissue samples from the TCGA-LIHC project was retrieved from the TCGA database (http://tcga.cancer.gov/; November 19, 2023). Clinical information of the TCGA-LIHC patients was sourced from the cBioPortal database (https://www.cbioportal.org/; November 22, 2023). Additionally, we accessed the preprocessed projects (HCCDB18, HCCDB25, and HCCDB30) from HCCDB as external validation sets (http://lifeome.net:809/#/home; January 13, 2024). These projects house bulk RNA-seq data and corresponding clinical information of patients. HCCDB18 was obtained from the ICGC-LIRI-JP project (https://dcc.icgc.org/). HCCDB25 was obtained from project OEP000321 (https://www.biosino.org/node/). HCCDB30 was obtained from project GSE148355 (https://www.ncbi.nlm.nih.gov/geo/). The HCC single-cell RNA-seq (scRNA-seq) datasets, GSE242889 and GSE149614, were obtained from the GEO database (https://www.ncbi.nlm.nih.gov/geo/; June 27, 2024). We acquired 5 tumor tissue samples from GSE242889 and 10 tumor tissue samples from GSE149614, respectively. The spatial transcriptomic data of HCC, HRA000437, were downloaded from the CNCB database (https://www.cncb.ac.cn/; January 15, 2024).

### 2.2 Data processing

Each patient in all datasets had only one tumor sample retained for analysis. In the TCGA-LIHC project, the original raw count data was employed for differential expression analysis, while Log2 (TPM+1) data was utilized for other analyses. We excluded redundant tumor samples from three recurrent sites of two patients in the TCGA dataset. Additionally, we excluded 29 tumor samples corresponding to patients with missing follow-up data or follow-up times less than 30 days. Furthermore, to mitigate the impact of rare HCC subtype gene expression pattern differences on subsequent analyses, we excluded 8 samples with pathology reports indicating “Hepatocellular Carcinoma plus Intrahepatic Cholangiocarcinoma” and “Fibrolamellar Carcinoma”. Ultimately, we included 334 tumor samples for further analysis. In the ICGC-LIRI-JP project, we excluded two patients with insufficient follow-up times, resulting in a total of 201 samples for subsequent analysis. We included all 158 patient samples from the OEP000321 project and 50 tumor samples with survival information from the GSE148355 project for analysis. The “Seurat” R package was utilized for processing 10× single-cell transcriptomic and spatial transcriptomic data. Quality control standards for scRNA-seq data included the following criteria: (1) nCount_RNA > 1000; (2) nFeature_RNA > 300; (3) percent_mito < 20%; (4) percent_ribo > 3%; (5) percent_hb < 0.1%. Subsequently, the “DoubletFinder” R package was used to eliminate potential doublets, and the “harmony” R package facilitated the integration of these fifteen samples. We utilized the uniform manifold approximation and projection (UMAP) for dimensionality reduction and visualization. Next, the “FindNeighbors” and “FindClusters” functions were employed for cell clustering. Manual annotation was performed to label different cell clusters. After normalizing the spatial transcriptomic data with the “SCTransform” function, we performed principal component analysis and UMAP dimensionality reduction. Subsequently, we utilized the “FindTransferAnchors” function to map the annotated information from the processed scRNA-seq data to the spatial transcriptomic data.

### 2.3 Identification of MVI-related genes in malignant cells

We isolated the epithelial cell population from all single-cell clusters and reclassified them. The “infercnv” R package was utilized to infer chromosomal copy number variation (CNV) of macrophages, T/NK cells, and epithelial cells in scRNA-seq data (23). We employed the “FindMarkers” function to identify differentially expressed genes in malignant cells from MVI-positive and MVI-negative samples. Specifically, genes with | avg_log2FC | > 1 and p_val_adj < 0.05 were designated as MVIRGs in malignant cells.

### 2.4 Analysis of prognosis-related single cells and MVI-related malignant cells

We utilized the “Scissor” R package (24) to predict and visualize single cells potentially associated with prognosis and malignant cells potentially associated with MVI, respectively. This was based on the gene expression patterns of tumor tissue and clinical information of patients from TCGA-LIHC. The “FindMarkers” function was applied to discern specifically expressed genes between Scissor+ and Scissor- cells within distinct cell clusters, as well as MVI_Scissor+ and MVI_Scissor- cells.

### 2.5 Pseudotime analysis of malignant cells

We conducted pseudotime analysis on malignant cells using the “Monocle” R package [8]. The “CytoTRACE” R package was utilized to aid in determining the direction of cell differentiation. By integrating the predictions from the Scissor algorithm, we conducted a comprehensive analysis of the differential differentiation trajectories between Scissor+ and Scissor- malignant cells, as well as between MVI_Scissor+ and MVI_Scissor- malignant cells.

### 2.6 Analysis of function and pathway enrichment

To gain a better understanding of the functional characteristics of malignant cell subpopulations, we utilized the “clusterProfiler” R package (25) to conduct Gene Ontology (GO) enrichment analysis on different states of malignant cells. Additionally, we employed the Gene Set Enrichment Analysis (GSEA) software (v4.3.2) along with its accompanying Hallmark gene sets to analyze the enriched signaling pathways among MVI-related malignant cell subgroups, and visualized the results using the “GseaVis” R package.

### 2.7 Cell-cell communication

We utilized the “CellChat” R package (26) to conduct intercellular interaction analysis in both single-cell transcriptomic data and spatial transcriptomic data. Our focus was on identifying differentially expressed signaling genes between malignant cells and other cell types and predicting the probability of intercellular communication as well as the associated pathways.

### 2.8 Construction, validation, and evaluation of the prognostic model

Using the “caret” R package, we randomly partitioned the tumor tissue samples from TCGA-LIHC in a 4:1 ratio to create training and testing sets. The ICGC-LIRI-JP project, OEP000321 project, and GSE148355 project are used for external validation. Due to the varying numbers of genes in different datasets (26729 genes in TCGA; 21362 genes in ICGC; 35690 genes in OEP000321; and 21444 genes in GSE148355), we intersected the MVIRGs with all datasets for further analysis. Univariate Cox regression analysis was used for preliminary screening for prognostic genes in the TCGA-LIHC training set.

Based on the study by Liu et al., we utilized 101 combinations of 10 machine-learning algorithms to construct a prognostic model. These algorithms included CoxBoost, elastic network (Enet), generalized boosted regression modeling (GBM), least absolute shrinkage and selection operator (Lasso), partial least squares regression for Cox (plsRcox), Ridge, random survival forest (RSF), stepwise Cox, supervised principal components (SuperPC), and survival support vector machine (survival-SVM). These combinations were applied to the selected representative MVIRGs, and LOOCV was employed to prevent overfitting. After the machine learning filtering of key variables, we utilized multivariate Cox regression to develop a prognostic model, as it facilitates model interpretation and practical implementation. The computational formula for the risk model is as follows:


$$risk\;score=\sum_{x=1}^{n}\left(coef\left(mRNAx\right)\times expr\left(mRNAx\right)\right)$$

coef (mRNAx) and expr (mRNAx) are the survival correlation coefficient and expression of MVIRGs involved in the construction of the model, respectively. For each model, Harrell’s concordance index (C-index) was calculated for the training, testing, and validation sets. The model with the highest average C-index was deemed optimal. Subsequently, we collected 37 published prognostic models for HCC and applied them to our TCGA-LIHC training set, internal testing set, and external validation sets to calculate the C-index. This facilitated a comparison between our study’s optimal model and the existing models.

Within the TCGA-LIHC training set, internal testing set, and external validation sets, patients were stratified into high- and low-risk groups based on the median risk score, respectively. The “survminer” R package was utilized to plot the survival curves corresponding to OS and DFS. After excluding patients with missing clinical information, the “pec” R package was used to visualize the time-dependent C-index for the risk score and clinical information. Additionally, the “rms” R package was employed to construct calibration curves. Furthermore, decision curve analysis (DCA) was developed in all the datasets to assess the guidance significance of the risk score for clinical decision-making in a 2-year timepoint.

### 2.9 Statistical analysis

All statistical analyses were performed using R language (v4.2.2). Univariate Cox regression was employed to screen MVIRGs associated with OS. The prognostic model and decision model for DCA comparison are based on multivariate Cox regression. Kaplan-Meier method was utilized to estimate the OS rates and DFS rates of patients stratified into high- and low-risk groups, and their significance was assessed using the log-rank test. A statistical significance threshold of p-value < 0.05 was applied to all statistical tests.

## 3 Results

### 3.1 Cell annotation

**Figure 1:**
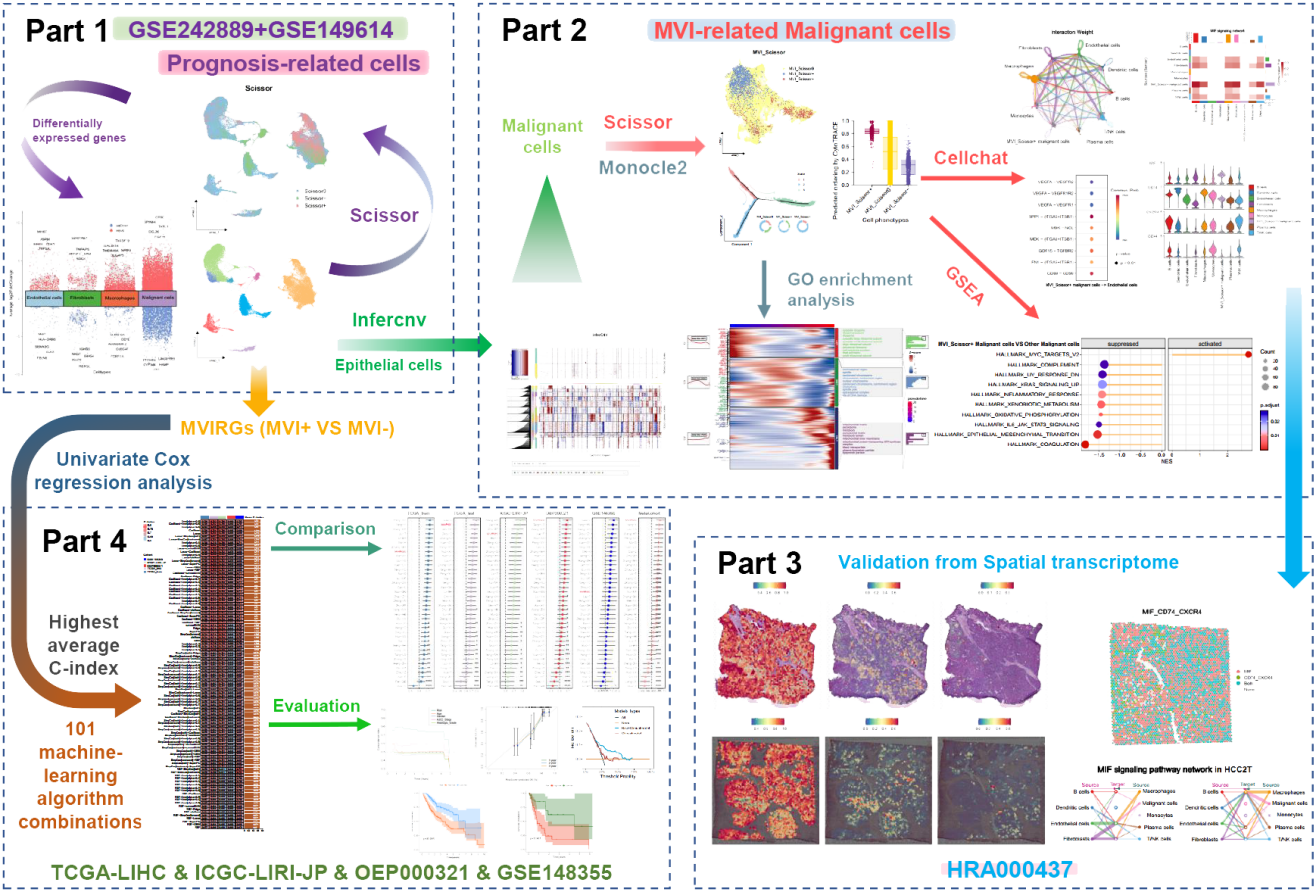
The overall flow chart of this study.

**Figure 2:**
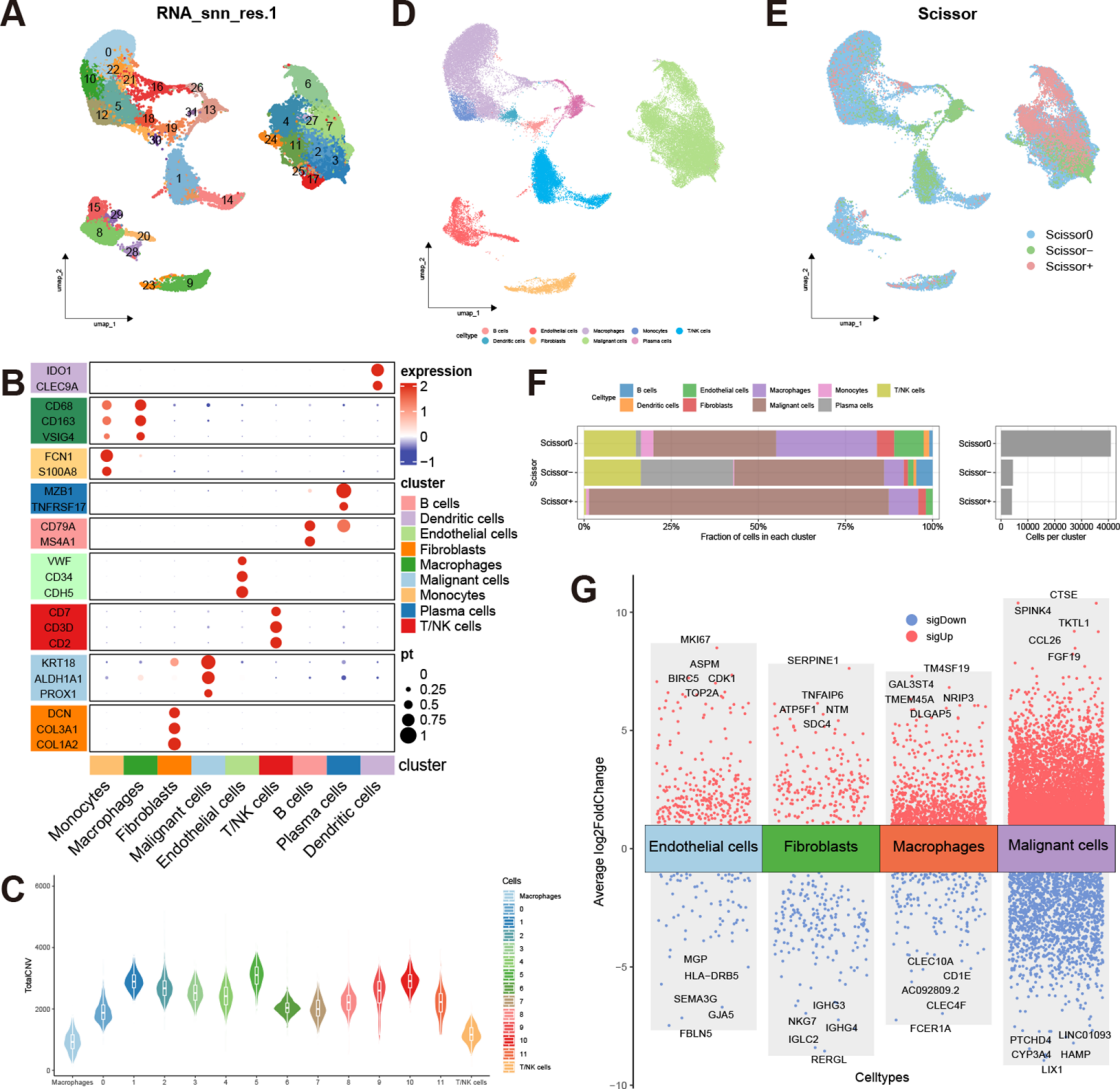
Cell annotation and analysis of prognosis-related cells. **(A)** UMAP distribution of 32 clusters at a resolution of 1; **(B)** Expression of markers corresponding to the 9 cell types (Mac, Macrophage; Mono, Monocyte; DC, Dendritic cell; B, B cell; Pla, Plasma cell; T_NK, T/NK cell; Epi, Epithelial cell; Fibro, Fibroblast; Endo, Endothelial cell); **(C)** Copy number variation scores of epithelial cells, macrophages, and T/NK cells; **(D)** UMAP distribution of 9 annotated cell types; **(E)** UMAP distribution of prognosis-related cells predicted by the Scissor algorithm; **(F)** Proportions of different cell types in Scissor0, Scissor-, and Scissor+ cells; **(G)** Volcano plot of differentially expressed genes in Scissor+ cells versus Scissor- cells within ECs, fibroblasts, macrophages, and malignant cells.

**Figure 1** shows the overall process of our study. After conducting quality control and removing doublets, a total of 50477 cells were included in the analysis. Using the “harmony” R package for sample integration, batch effects were effectively mitigated (**Supplementary Figure S1A**). Employing the “clustree” function, we showcased cell clustering at various resolutions, ultimately opting for a resolution of 1 to partition all cells into 32 distinct subgroups (**Figure 2A**, **Supplementary Figure S1B**). Subsequently, we manually annotated different cell clusters, drawing from established literature (**Figure 2B**). The markers used to delineate various cell clusters in this study are as follows: macrophages (CD163, CD68, VSIG4); monocytes (S100A8, FCN1); dendritic cells (CLEC9A, IDO1); B cells (MS4A1, CD79A); plasma cells (MZB1, TNFRSF17); T/NK cells (CD2, CD3D, CD7); epithelial cells (KRT18, PROX1, ALDH1A1); fibroblasts (COL3A1, COL1A2, DCN); endothelial cells (VWF, CDH5, CD34) (27–32). Notably, due to the co-expression of markers of different cell types in clusters 21, 25, and 28, suggesting potential doublets, these cells were excluded from further analysis. Subsequently, we isolated the epithelial cell cluster and re-clustered it into 12 subgroups using a resolution of 0.1. The “infercnv” R package was then employed to compute the CNV scores for each epithelial cell subgroup, allowing for the discrimination of malignant epithelial cells. The results revealed a significant increase in CNV across all epithelial cell subgroups compared to macrophages and T/NK cells, indicating the malignant nature of these epithelial cells (**Figure 2C**, **Supplementary Figure S1C**). Based on distinct cell markers, we initially categorized all cells into 9 types (**Figure 2D**).

### 3.2 Analysis of prognosis-related single cells

The Scissor algorithm has been proven to be a highly accurate predictive tool (24, 33). Using TCGA-LIHC data as input, we employed survival prognosis to predict potential prognosis-related single cells within the single-cell data. The alpha parameter was set at 0.02, and we got 4073 Scissor+ cells and 4441 Scissor- cells. The predictive results were validated through 10-fold cross-validation, yielding a p-value of less than 0.001 (**Supplementary Figure S1D, E**). Scissor+ represents an association with an unfavorable prognosis, while Scissor- indicates an association with favorable prognosis, and Scissor0 denotes no significant correlation (**Figure 2E**). The largest number of prognosis-related single cells were found within the malignant cells. Interestingly, T/NK cells, fibroblasts, macrophages, and endothelial cells (ECs) also exhibited the presence of cells associated with unfavorable prognosis. Conversely, B cells and plasma cells were predominantly associated with a favorable prognosis (**Figure 2F**). In addition to the malignant cells, we analyzed differentially expressed genes between Scissor+ cells and Scissor- cells within macrophages, fibroblasts, and ECs, respectively (**Supplementary Table S1–S4**). The “scRNAtoolVis” R package was utilized to generate volcano plots displaying the top ten differentially expressed genes for each cell population (**Figure 2G**). We observed that within the three subgroups associated with adverse prognosis, TUBB, TUBA1B, and ENO1 were all overexpressed by more than 2-fold, indicating potentially heightened metabolic and migratory capabilities of these cells. Furthermore, the Scissor+ malignant cells, macrophages, and ECs also exhibit high expression of cell cycle-related genes such as TOP2A, MKI67, and PCNA, indicating the high proliferative capacity of these cell populations.

**Figure 3:**
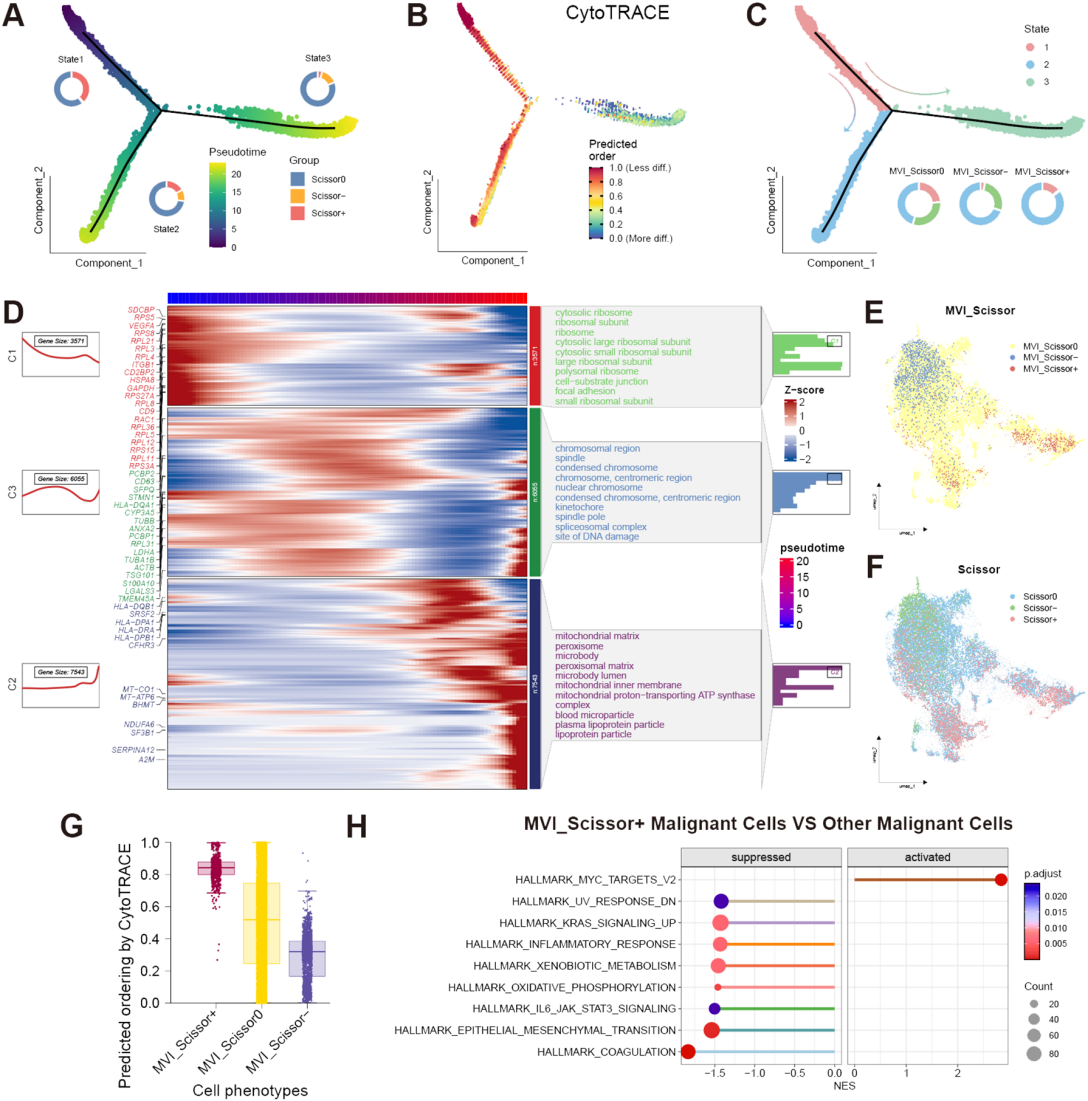
Analysis of malignant cells. **(A)** Proportion of Scissor0, Scissor-, and Scissor+ malignant cells in different states and pseudotime direction predicted by Monocle2; **(B)** Predicted differentiation direction of malignant cells by CytoTRACE; **(C)** Proportion of different states malignant cells in MVI_Scissor0, MVI_Scissor-, and MVI_Scissor+ groups; **(D)** Corresponding gene expression heatmaps and enriched Gene Ontology (GO) cellular components of three malignant cell clusters related to pseudotime; **(E)** UMAP distribution of MVI-related malignant cells predicted by the Scissor algorithm; **(F)** UMAP distribution of prognosis-related malignant cells predicted by the Scissor algorithm; **(G)** CytoTRACE scores of MVI_Scissor0, MVI_Scissor-, and MVI_Scissor+ malignant cells; **(H)** Differential hallmark pathway enrichments between MVI_Scissor+ malignant cells and other malignant cells analyzed by GSEA.

To further investigate the differentiation trajectory of malignant cells, we conducted a pseudotime analysis. The results indicated that malignant cells can be categorized into three states through a single intersection point. The Monocle2 software package suggested that state-1 malignant cells represent the initiation of differentiation, while state-3 represents the terminal differentiation. Scissor+ malignant cells were predominantly located at the early and middle stages of differentiation, whereas Scissor- malignant cells were mainly found at the terminal stages of differentiation. We further validated these findings using the “CytoTRACE” R package (**Figures 3A–C**). Additionally, by employing the “ plot_pseudotime_heatmap2” function of the “ClusterGVis” R package, we classified malignant cells into 3 cell clusters and analyzed the cellular component genes enriched in these cell clusters (**Figure 3D**). From the enrichment analysis, it was evident that the malignant cell cluster C1 located at the initiation of differentiation was highly enriched in ribosome-related genes, indicating its potential robust biosynthetic capacity. It is well-established that the malignant proliferative capacity of malignant cells relies on increased protein synthesis, which in turn depends on ribosomal complexes (34). Translation dysregulation is considered a hallmark of cancer stem cells (CSCs), with translational control also enhancing the plasticity of cancer cells, thereby promoting tumor progression and metastasis (35). The excessive activity of ribosome biogenesis is associated with advanced tumor staging and lower cancer survival rates, aligning with the predominance of Scissor+ malignant cells at the initiation of differentiation (36). Cluster C3 was enriched in chromosome and splicing-related genes. Recent studies have validated that mutations or abnormal expression of spliceosome-related genes are linked to tumor progression, with some of these genes also regulating immune signal transduction (37). Therefore, malignant cells in Cluster C1 and C3, located at the early to middle stages of differentiation, may both be associated with tumor progression and metastasis. Interestingly, malignant cells in these two clusters also exhibit high expression of exosome marker genes such as CD9, CD63, and TSG101. The exosome marker proteins were obtained from the ExoCarta database (http://www.exocarta.org; January 29, 2024). Extracellular vesicles play a crucial role in tumor metastasis, facilitating communication between malignant cells and other cells (38). Therefore, cells in clusters C1 and C3 may also play a pivotal role in influencing the TME. It is noteworthy that malignant cells in cluster C2 at the terminal stages of differentiation exhibit high expression of MHC-related genes such as HLA-DRA, HLA-DPB1, and HLA-DQB1. This expression pattern overlaps with the distribution of Scissor- malignant cells. Previous research has indicated that tumor-specific MHC expression can potentially elicit immune recognition of the tumor, thereby augmenting immune-mediated malignant cell destruction (39). This heightened MHC expression may contribute to the favorable prognosis associated with this specific cell cluster.

### 3.3 Analysis of MVI-related malignant cells

It becomes evident that not all malignant cells are directly associated with adverse prognoses, underscoring the heterogeneity of malignant cells. To delve deeper into the malignant cells associated with MVI, we employed the Scissor algorithm, substituting the phenotype with the occurrence of MVI in TCGA patients. We included 98 tumor samples from MVI-positive patients and 146 tumor samples from MVI-negative patients, setting the α parameter to 0.05, ultimately identifying 622 MVI_Scissor+ malignant cells and 1462 MVI_Scissor- malignant cells (**Supplementary Figure S1F, G**). MVI_Scissor+ represents an association with the MVI-positive phenotype, while MVI_Scissor- indicates an association with the MVI-negative phenotype, and MVI_Scissor0 denotes no significant correlation. Subsequent analysis revealed that, in comparison to the other malignant cells, the overall gene expression profile of MVI_Scissor+ malignant cells closely resembled Scissor+ malignant cells (**Figures 3E, F**, **Supplementary Table S5**). The MVI_Scissor+ malignant cells predicted by CytoTRACE scored the highest, meaning they were immature malignant cells (**Figure 3G**). Consequently, we hypothesize that the malignant cells leading to clinical MVI occurrence belong to the initial differentiation rather than the terminal differentiation of malignant cells.

Through GSEA analysis, we investigated the differential enrichment of signaling pathways in MVI_Scissor+ malignant cells compared to other malignant cells. | normalized enrichment score (NES) | > 1.4 and FDR q-value < 0.05 were considered screening conditions. The results revealed that MVI_Scissor+ malignant cells exhibited significant enrichment only in the HALLMARK_MYC_TARGETS_V2 pathway. In contrast, other malignant cells are involved in a wider range of biological activities, such as coagulation, epithelial-mesenchymal transition, oxidative phosphorylation, and processing of exogenous substances. This suggested that MVI_Scissor+ malignant cells possess unique functional characteristics, primarily associated with MYC-regulated cell proliferation (**Figure 3H**, **Supplementary Table S6**).

**Figure 4:**
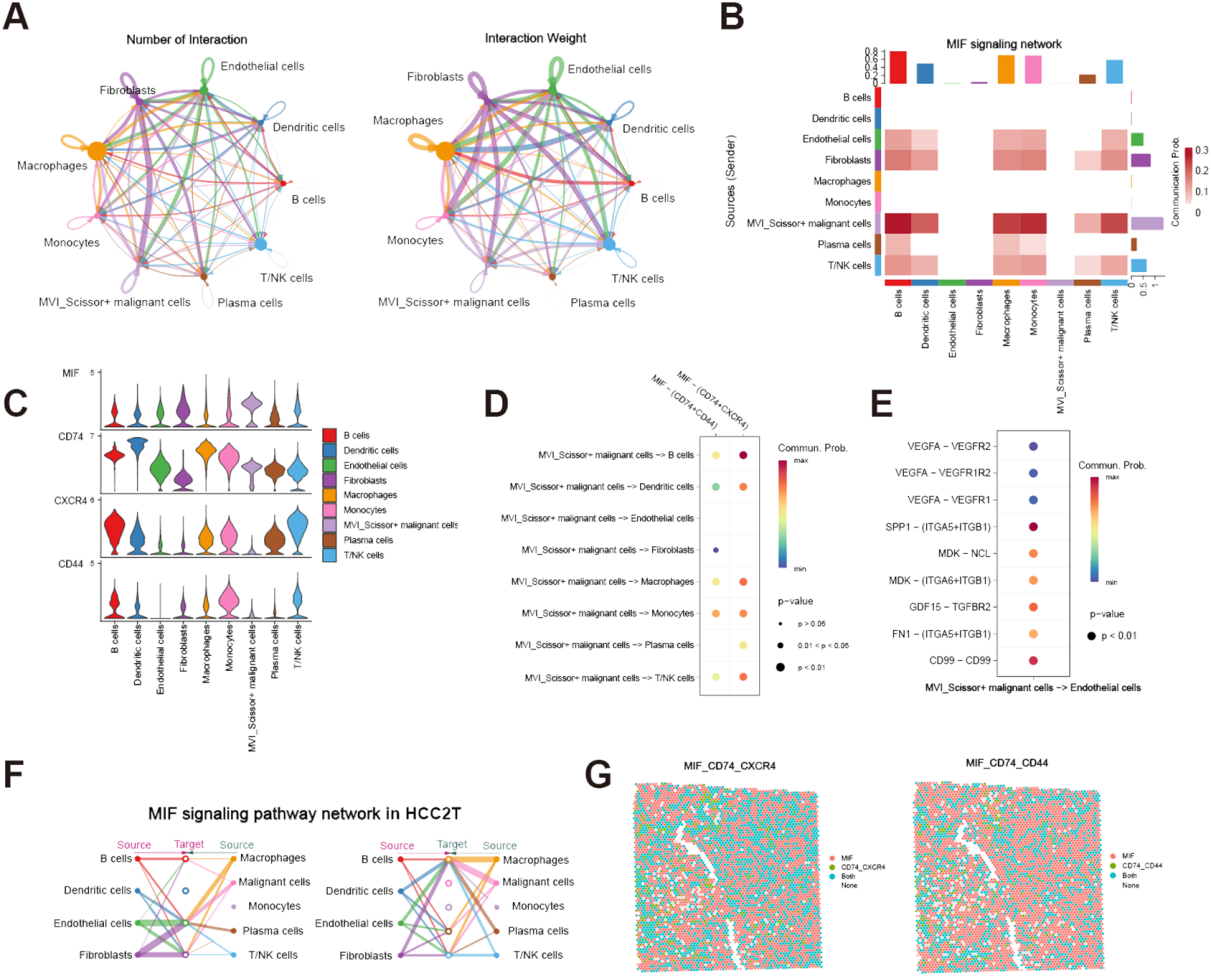
Cell-cell communication analysis of MVI-related malignant cells. **(A)** The number and weight of communications between MVI_Scissor+ malignant cells and other cell types; **(B)** Heatmap of MIF signaling pathway communications between MVI_Scissor+ malignant cells and other cell types; **(C)** Expression levels of MIF signaling pathway receptor-ligand pairs in different cell types; **(D)** Communication probabilities of various receptor-ligand pairs in the MIF signaling pathway between MVI_Scissor+ malignant cells and other cell types; **(E)** Comparison of predicted communication pathways between MVI_Scissor+ malignant cells and ECs; **(F)** Predicted communications of the MIF signaling pathway between malignant cells and other cells in HCC-2T; **(G)** Co-localization of MIF-(CD74+CXCR4) and MIF-(CD74+CD44) receptor-ligand pairs in HCC-2T.

To further investigate the role of MVI_Scissor+ malignant cells in the tumor immune microenvironment, we conducted a cell-cell communication analysis. The results indicate rich interactions between MVI_Scissor+ malignant cells and other cell types, with the MIF (macrophage migration inhibitory factor) signaling pathway being widely distributed among various cell populations (**Figures 4A, B**, **Supplementary Table S7**). The MIF signaling pathway encompasses two receptor pairs, and our analysis revealed that MIF-(CD74+CXCR4) exhibited the highest predicted probability and the most widespread distribution. However, the communication probability of MIF-(CD74+CD44) was not as high as MIF-(CD74+CXCR4) (**Figures 4C, D**). What’s more, the enrichment of the MIF pathway between MVI_Scissor+ malignant cells and ECs was not significant. Interestingly, the most significant signaling between MVI_Scissor+ malignant cells and ECs was through SPP1-(ITGA5+ITGB1), surpassing the VEGF signaling pathway (**Figure 4E**). Taken together, the MIF signaling pathway may represent a potential target for disrupting the interaction between MVI_Scissor+ malignant cells and other cell types.

### 3.4 Validation from the spatial transcriptome

We performed external validation using spatial transcriptomic data HCC-1 and HCC-2 from project HRA000437 of the CNCB database. HCC-1 samples were derived from tumor sections of patients without MVI, while HCC-2 samples were obtained from patients with MVI. In addition to the tumor (T) and the surrounding tissue (L), HCC-2 includes portal vein tumor thrombus samples (P) compared to HCC-1. Cell populations were annotated based on scRNA-seq data, yielding annotations basically consistent with those of Wu et al. (**Supplementary Figures S2A, B**, **Supplementary Figures S3A–C**). We conducted cell-cell communication analysis in the HCC-2T sample (**Supplementary Table S8**). The results indicated significant communication of malignant cells with macrophages, ECs, and fibroblasts via MIF signaling (**Figure 4F**). Furthermore, co-localization analysis of MIF and (CD74+CXCR4) or (CD74+CD44) affirmed the consistency and widespread nature of this receptor’s distribution (**Figure 4G**).

**Figure 5:**
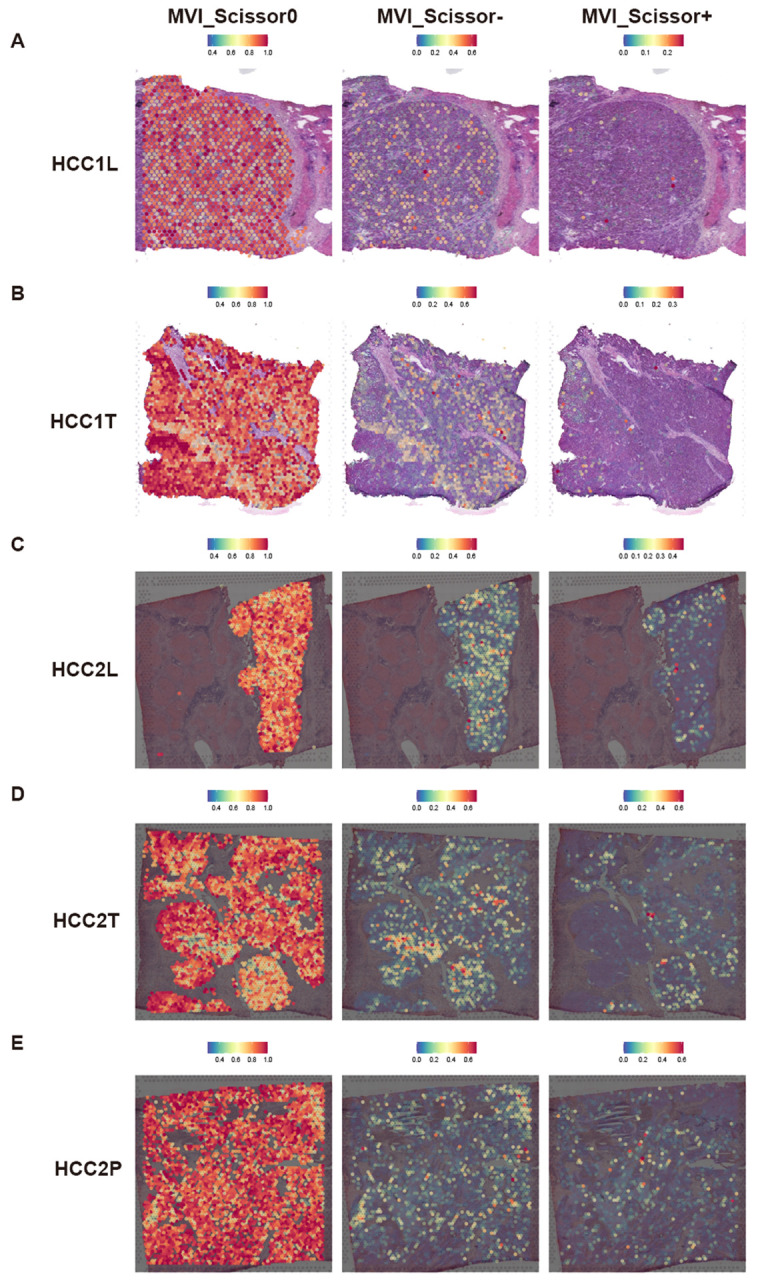
Predicted distribution of MVI-related malignant cells in spatial transcriptomic data. **(A–E)** Predicted distribution of MVI_Scissor0, MVI_Scissor-, and MVI_Scissor+ malignant cells in HCC-1L, HCC-1T, HCC-2L, HCC-2T, and HCC-2P, respectively.

Subsequently, we focus on the spatial distribution of malignant cells in different states and those predicted to be MVI-related and prognosis-related by the Scissor algorithm. Our analysis revealed that, regardless of MVI status, Scissor0 and MVI_Scissor0 malignant cells predominated. Conversely, Scissor-, and MVI_Scissor- malignant cells were more abundant in tumors without MVI, while Scissor+ and MVI_Scissor+ malignant cells were notably more prevalent in MVI-positive tumors. This validates the accuracy of our prediction of MVI-related malignant cells (**Figures 5A–E**, **Supplementary Figures S4A–E**).

### 3.5 Construction, validation, and evaluation of the prognostic model

**Figure 6:**
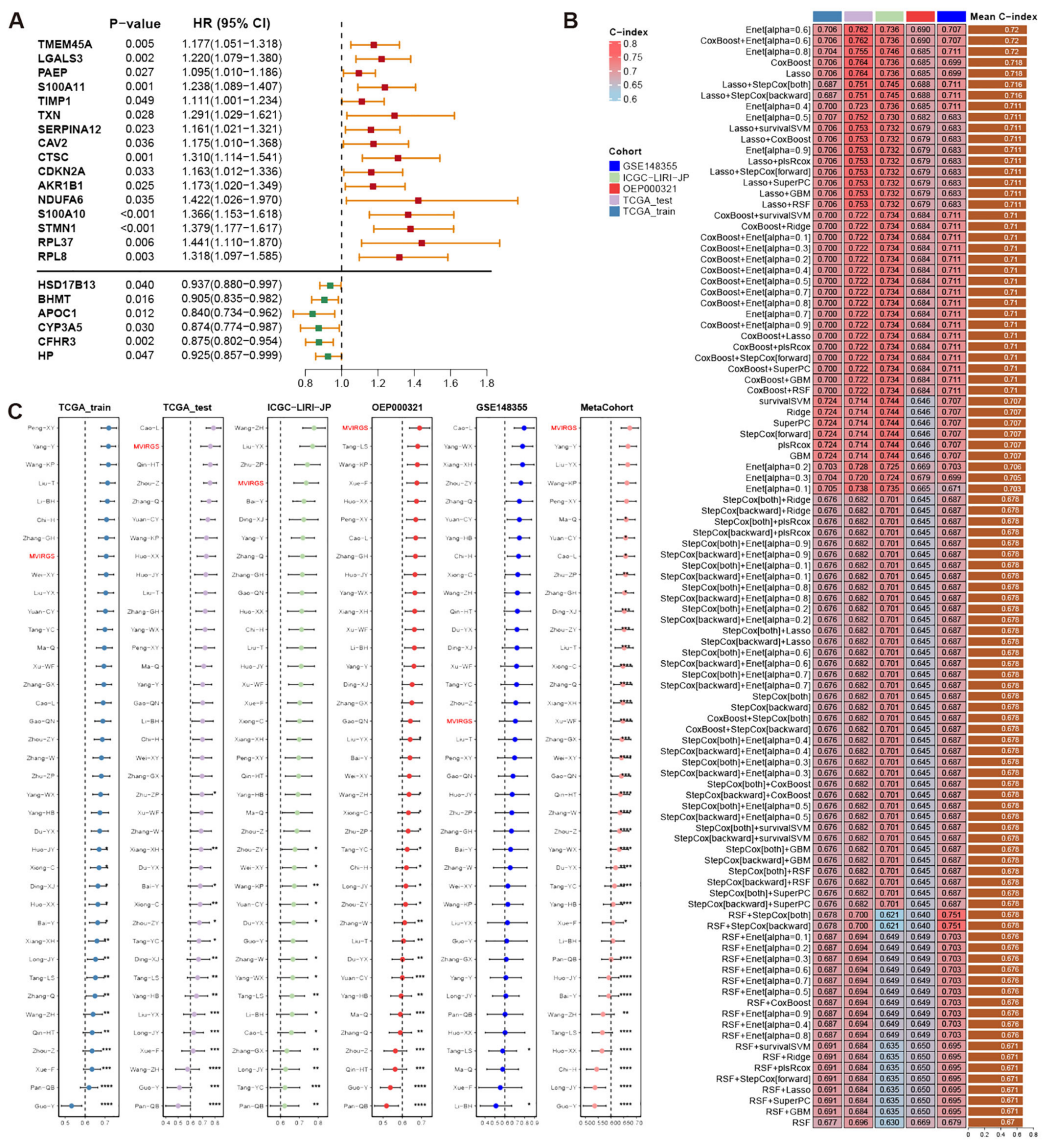
Construction, validation, and comparison of prognostic model based on MVIRGs. **(A)** Representative MVIRGs with statistically significant results from univariate Cox regression analysis in the TCGA-LIHC training set; **(B)** C-index of different models constructed using 101 machine-learning algorithm combinations in the TCGA-LIHC training set, testing set, and external validation sets; **(C)** C-index comparison of the prognostic model based on MVIRGs and 37 published HCC prognostic models in the TCGA-LIHC training set, testing set, and external validation sets.

To investigate tumor heterogeneity in MVI-positive and MVI-negative patients, we identified MVI-related genes within malignant cells, which are provided in **Supplementary Table S9**. These genes were applied to construct a prognostic model. In the TCGA-LIHC training set, the results from univariate Cox regression analysis revealed significant associations between the expression levels of 38 MVIRGs and OS. Specifically, 23 genes were correlated with an unfavorable prognosis, while 15 genes were correlated with a favorable prognosis (**Supplementary Table S10**). Genes exhibiting high expression and a Hazard ratio (HR) greater than 1 in malignant cells of MVI-positive patients, as well as those demonstrating high expression and an HR less than 1 in malignant cells of MVI-negative patients, were selected (**Figure 6A**). These 22 genes were designated as representative MVIRGs and were subsequently incorporated into the modeling process.

After identifying 22 representative MVIRGs, we utilized 101 combinations of 10 machine-learning algorithms to individually screen for distinct key genes. These genes were then used to build 101 multivariate Cox regression models. The C-indexes of these 101 models were calculated in the TCGA-LIHC training set, internal testing set, and external validation sets (**Figure 6B**). The optimal model was the Enet (alpha=0.6) model, achieving an average C-index of 0.72. This model comprised 10 genes: TMEM45A, S100A10, NDUFA6, STMN1, SERPINA12, LGALS3, BHMT, CYP3A5, CFHR3, and RPL8. The final formula for the risk model is as follows:


$$\begin{array}{l} Risk\;score=\;0.12568178\;\times \;TMEM45A\;+\\ \;0.07016186\;\times \;S100A10\;+\;0.14298943\times \;NDUFA6\;\\ +\;0.13330516\;\times \;STMN1\;+\;0.08159631\;\times \;\\ SERPINA12\;+\;0.06043725\;\times \;LGALS3\;\\ +\;0.15569238\;\times RPL8-0.01400613\;\times \;BHMT\;-\;\\ 0.05863893\;\times \;CYP3A5-0.09699672\;\times \;CFHR3 \end{array}$$

Differential expression analysis of TCGA-LIHC tumor tissues and adjacent normal tissues revealed that the expression differences of TMEM45A, S100A10, SERPINA12, BHMT, CFHR3, RPL8, and STMN1 all exceed a 2-fold change (**Supplementary Table S11**). The findings indicated the potential significance of these genes.

**Figure 7:**
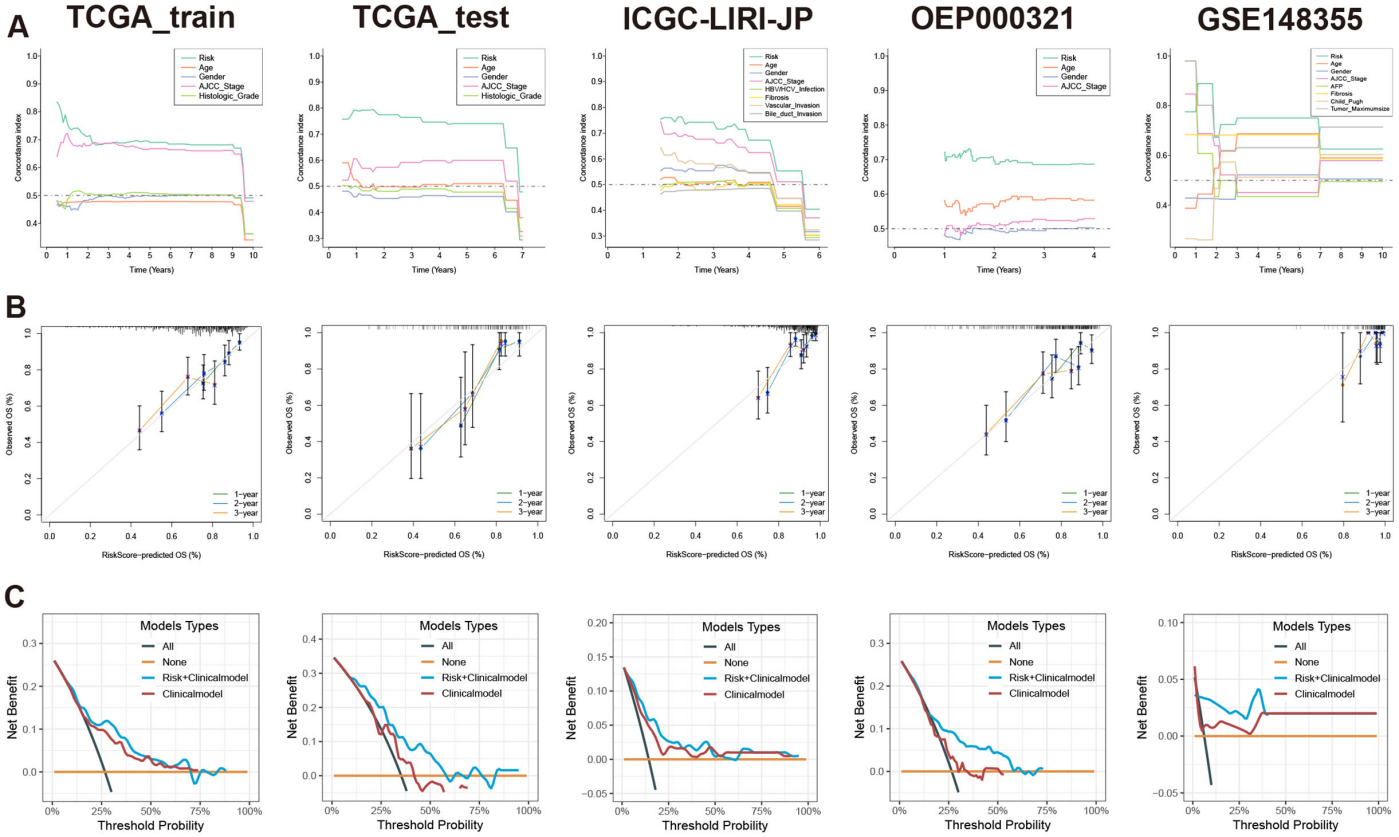
Evaluation of prognostic model based on MVIRGs using time-dependent C-index curves, calibration curves, and DCA. **(A)** Comparison of time-dependent C-index curves based on the risk model and single clinical information in all the datasets; **(B)** 1-, 2-, and 3-year calibration curves of the prognostic model in the TCGA-LIHC training set, testing set, and external validation sets; **(C)** Comparison of 2-year DCA between the multivariate Cox model constructed using risk scores combined with clinical information and the multivariate Cox model constructed using only clinical information in all the datasets.

When compared with 37 published HCC prognostic models, our selected risk model demonstrated significant stability (**Figure 6C**, **Supplementary Table S12**). We evaluated the sensitivity and specificity of the prognostic predictions by plotting time-dependent C-index curves. After excluding clinical information with significant missing data across various datasets, the clinical information included in our analysis was as follows: the TCGA-LIHC dataset included age, gender, AJCC (American Joint Commission of Cancer) stage, and histologic grade; the ICGC-LIRI-JP dataset included age, gender, AJCC stage, HBV/HCV infection status, liver fibrosis grade, vascular invasion status, and bile duct invasion status; the OEP000321 dataset included age, gender, and AJCC stage; the GSE148355 dataset included age, gender, AJCC stage, AFP (Alpha-fetoprotein) levels, liver fibrosis grade, Child-Pugh grade, and maximum tumor size. Across all datasets, the risk model demonstrated excellent performance by comparing the predictive efficacy of individual clinical information (**Figure 7A**). Additionally, calibration curves for 1-, 2-, and 3-year periods confirmed that the risk model’s predicted OS closely mirrored actual outcomes (**Figure 7B**). To assess the model’s impact on clinical decision-making, we performed DCA at a 2-year timepoint. These curves included two multivariate Cox regression models: one based on clinical information mentioned above, and the other combining risk scores with clinical information. Across all datasets, the risk-combined clinical model yielded greater net benefit for patients compared to the clinical model alone. For instance, in the TCGA_test dataset, the net benefit of the predictive strategy based on the risk-combined clinical model significantly exceeded that of the clinical model predictive strategy, with threshold probabilities ranging from approximately 10% to 60% (**Figure 7C**).

**Figure 8:**
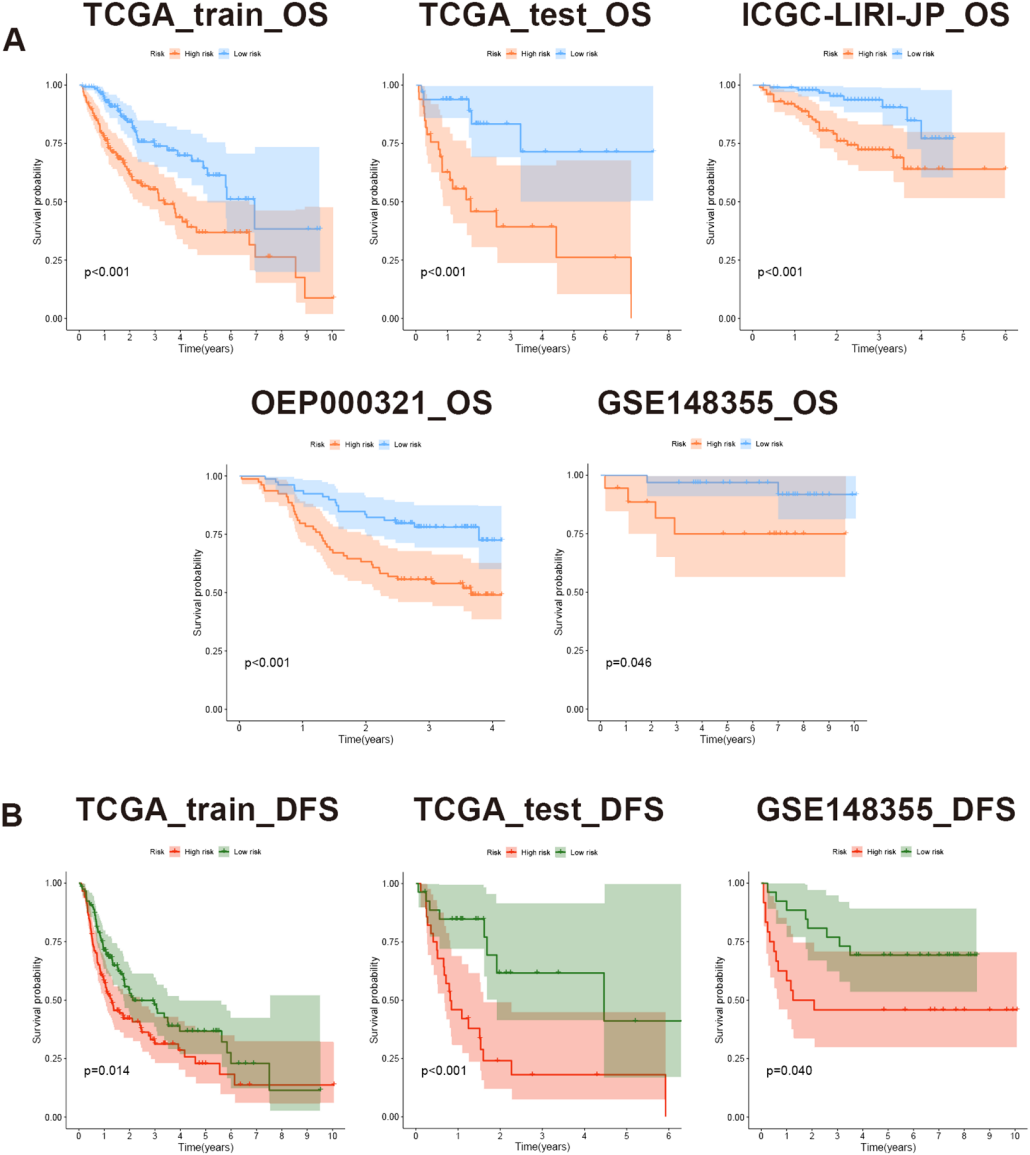
Evaluation of prognostic model based on MVIRGs using survival curves. **(A)** OS differences between high- and low-risk groups in all the datasets; **(B)** DFS differences between high- and low-risk groups in the TCGA-LIHC training set, testing set, and GSE148355 validation set.

Based on the risk scores, patients were stratified into high-risk and low-risk groups equally. To assess differences in survival time and status between the two groups, survival curves were constructed. The result clearly illustrated that the high-risk group displayed a significantly unfavorable prognosis compared to the low-risk group. Moreover, across the training, testing, and validation sets, all log-rank test P-values were below 0.05, indicating notable disparities (**Figure 8A**). Importantly, the model not only discriminated between high- and low-risk patients in terms of OS but also revealed remarkably significant differences in DFS (**Figure 8B**).

## 4 Discussion

Previous studies have highlighted the pivotal role of MVI in the postoperative recurrence of hepatocellular carcinoma, often associated with heightened tumor invasiveness. As the extent of MVI increases, such as a rise in MVI quantity and its distance from the main tumor, the prognosis for patients becomes increasingly unfavorable (11, 40). The diagnosis of MVI typically relies on histopathological microscopic examination of resected surgical specimens, limiting the potential for early detection. Given this scenario, an increasing number of researchers are exploring methods, including radiomics, to predict the presence of MVI preoperatively (41–44). However, even upon identifying the likelihood of MVI, there remains a lack of systematically targeted treatment strategies, despite some studies reporting potential considerations, such as postoperative adjuvant hepatic arterial infusion chemotherapy with 5-fluorouracil and oxaliplatin (45, 46). This is due to the unclear mechanisms underlying the occurrence and progression of MVI. Therefore, delving into the specific biological origins of MVI becomes particularly crucial.

In this study, we comprehensively analyzed the biological characteristics of MVI by integrating bulk RNA-seq, single-cell RNA-seq, and spatial transcriptomics data containing clinical information on MVI from public databases. The invention of the Scissor algorithm has enabled the prediction of single-cell subpopulations associated with clinical phenotypes, facilitating relevant studies on the origin and development of diseases (24). Utilizing this algorithm, we predicted prognosis-related single cells and MVI-related malignant cells and validated the accuracy of these predictions using spatial transcriptomics data. Through combined pseudo-time analysis and intercellular communication analysis, we have proposed a novel hypothesis: the occurrence and progression of MVI are determined by the distinct differentiation fates of tumor cells. The presence of tumor cells located at the early and middle stages rather than the terminal stage of differentiation correlates with a higher likelihood of microvascular invasion. In the terminal stage of differentiation, some tumor cells exhibit high expression of MHC-related proteins, potentially rendering them more susceptible to recognition and elimination by immune cells. Consequently, these tumor cells may exert an opposing effect on the occurrence and progression of MVI and unfavorable prognosis. Importantly, the proportion of these malignant cells potentially impacts the efficacy of immunotherapy and prognosis to a certain extent. Conversely, MVI-positive patients harbor a higher proportion of MVI_Scissor+ malignant cells, thus promoting MVI progression and leading to an unfavorable prognosis.

Interestingly, MVI-related malignant cells do not enrich pathways related to metabolism but rather in the MYC pathway. This indicates that the biological behavior of MVI_Scissor+ malignant cells is largely regulated by the MYC transcription factor. Conducting in-depth research on MYC downstream targets may be an effective direction to inhibit this subgroup and potentially impede the progression of MVI. In terms of intercellular communication, we observed minimal signals emitted by other cells towards MVI_Scissor+ malignant cells, with only partial pathways from fibroblasts and ECs. Conversely, MVI_Scissor+ malignant cells emit a substantial amount of signals to other cell types, particularly through the MIF signaling pathway. The receptors involved in the MIF signaling pathway exhibit the highest and most widespread interaction strength with MIF-(CD74+CXCR4). Furthermore, widespread co-localization of MIF-(CD74+CXCR4) was observed in spatial transcriptomics, suggesting that the MIF signaling pathway may be a key mechanism through which MVI_Scissor+ malignant cells exert significant influence in the tumor microenvironment. In conclusion, further in-depth research is warranted to elucidate the role of MIF in the occurrence and progression of MVI.

In addition to tumor cells, our investigation also focused on prognosis-associated macrophages, fibroblasts, and ECs. Macrophages linked to unfavorable prognosis exhibited heightened expression of SPP1, TREM2, LGALS3, CTSB, CTSD, CTSL, and FABP5, indicative of a lipid-related macrophage phenotype (47). Consistent with prior findings, these macrophages have been implicated in promoting malignant cell epithelial-mesenchymal transition and exerting immunosuppressive effects (32, 48). The fibroblasts associated with poor prognosis exhibit high expression of extracellular matrix-related markers, including COL5A1, COL6A3, POSTN, LUM, DCN, and FAP, consistent with the mCAFs defined by Zhu et al. This subgroup may be associated with epithelial-mesenchymal transition, hence positively correlated with poor prognosis (49). Furthermore, ECs associated with poor prognosis show high expression of LYVE1 and CD36, suggesting that this subgroup belongs to liver sinusoidal ECs (50).

The AJCC staging system is commonly used in clinical practice to assess patient prognosis. However, the heterogeneity of tumors makes predicting OS and DFS challenging. Shindoh et al. demonstrated that optimizing the prognostic assessment of the eighth edition AJCC staging system by incorporating patient MVI status underscores the importance of MVI (51). Through validation using training, testing, and external validation sets, we found that the model constructed based on MVIRGs demonstrates good generalizability and outperforms conventional AJCC staging systems and most previously published gene models. In future clinical applications, consideration could be given to integrating our model with Shindoh et al.’s new AJCC staging system to comprehensively evaluate patient prognosis from anatomical, pathological, and transcriptomic perspectives. By sequencing tumor samples from patients, we can calculate the risk scores for each patient based on the risk model formula. Suitable risk score threshold values will be identified based on a sufficiently large sample size. Additionally, incorporating more prognostic clinical information, and constructing a nomogram to refine patient scoring, thereby predicting overall survival time.

Admittedly, our study has certain limitations due to the relatively small sample size. Currently, there is limited inclusion of MVI as a patient stratification factor in single-cell studies of hepatocellular carcinoma (HCC). We aim to collect more data in this direction in the future. To mitigate bias, we integrated bulk, single-cell, and spatial transcriptomic data from different centers for multi-omics validation. Therefore, we believe in the high reliability of our results. Nevertheless, further *in vivo* and *in vitro* experiments are still needed to validate and expand our findings, especially regarding the molecular pathway mechanisms involved in the biological functions of MVI-related malignant cells.

In conclusion, our study identified a malignant cell subgroup related to MVI in HCC. By analyzing differentiation and developmental trajectories as well as intercellular interactions, we elucidated the primary biological functional characteristics of this subgroup. Moreover, we utilized a machine learning algorithm to construct a prognostic model based on MVIRGs. Our model exhibited excellent predictive performance and holds the potential to assist in clinical decision-making, thereby offering substantial benefits for patients. This provides new directions and insights for the treatment of HCC patients with MVI in the future.

## Data availability statement

The original contributions presented in the study are included in the article/**Supplementary Material**. Further inquiries can be directed to the corresponding author.

## Author contributions

HH: Conceptualization, Data curation, Formal analysis, Methodology, Visualization, Writing – original draft, Writing – review & editing. FW: Conceptualization, Data curation, Writing – original draft. YY: Conceptualization, Writing – original draft. BX: Writing – original draft, Formal analysis. DC: Writing – original draft, Data curation. YH: Data curation, Writing – original draft. SL: Writing – review & editing, Funding acquisition, Methodology, Project administration, Resources, Supervision, Validation.

## Supplementary material

Supplementary Figure 1 Results of scRNA-seq data processing. **(A)** UMAP visualization of 15 tumor samples from GSE242889 and GSE149614 before and after integration using harmony; **(B)** Single-cell clustering analysis at different resolutions using the “clustree” function; **(C)** Comparison of copy number variation in epithelial cell subtypes, macrophages, and T/NK cells analyzed using the “infercnv” R package; **(D, E)** Results of prognosis-relate single-cell prediction and 10-fold cross-validation using the Scissor algorithm; **(F, G)** Results of MVI-related malignant cell prediction and 10-fold cross-validation using the Scissor algorithm.

Supplementary Figure 2 Predicted distribution of different cell types in spatial transcriptomic data of MVI-negative Patients. **(A, B)** Predicted distribution of 9 different cell types in HCC-1L and HCC-1T, respectively.

Supplementary Figure 3 Predicted distribution of different cell types in spatial transcriptomic data of MVI-positive Patients. **(A–C)** Predicted distribution of 9 different cell types in HCC-2L, HCC-2T, and HCC-2P, respectively.

Supplementary Figure 4 Predicted distribution of prognosis-related malignant cells in spatial transcriptomic data. **(A–E)** Predicted distribution of Scissor0, Scissor-, and Scissor+ malignant cells in HCC-1L, HCC-1T, HCC-2L, HCC-2T, and HCC-2P, respectively.
