# Supplementary figures and images for: Multi-transcriptomics analysis of microvascular invasion-related malignant cells and development of a machine learning-based prognostic model in hepatocellular carcinoma
Source: Front Immunol. 2024 Aug 8;15:1436131. doi: 10.3389/fimmu.2024.1436131 (PMC11338809; doi:10.3389/fimmu.2024.1436131)

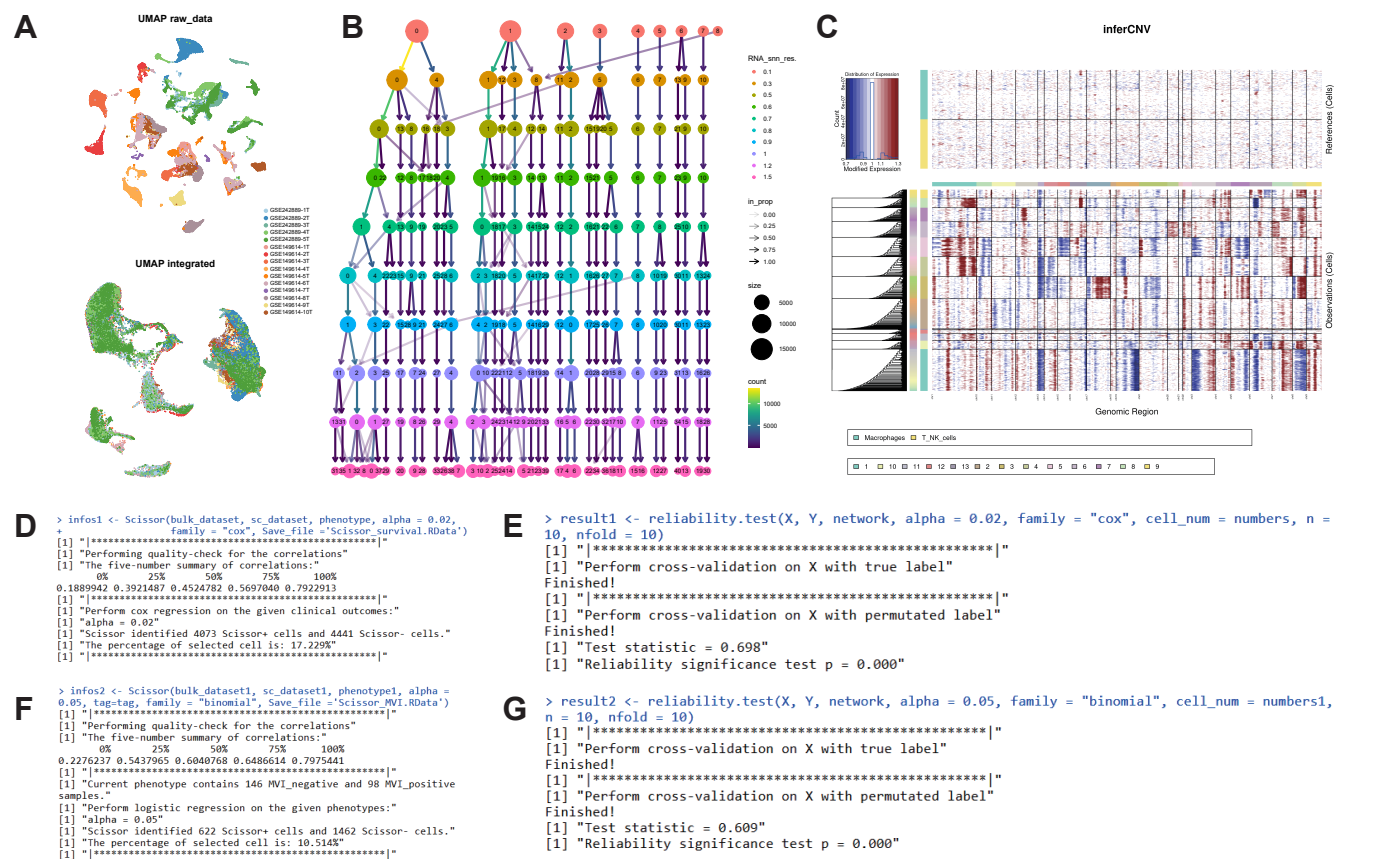

Supplement: Supplementary Figure 1 — Results of scRNA-seq data processing. (A) UMAP visualization of 15 tumor samples from GSE242889 and GSE149614 before and after integration using harmony; (B) Single-cell clustering analysis at different resolutions using the “clustree” function; (C) Comparison of copy number variation in epithelial cell subtypes, macrophages, and T/NK cells analyzed using the “infercnv” R package; (D, E) Results of prognosis-relate single-cell prediction and 10-fold cross-validation using the Scissor algorithm; (F, G) Results of MVI-related malignant cell prediction and 10-fold cross-validation using the Scissor algorithm. [file Image_1.pdf]

# Celltypes

**A**

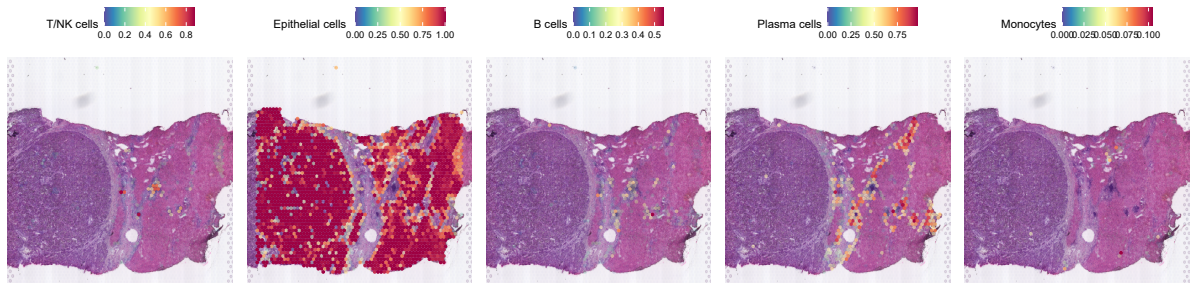

**HCC1L**

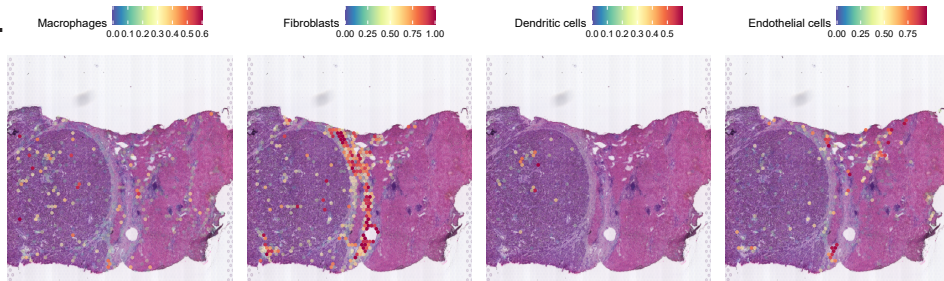

**B**

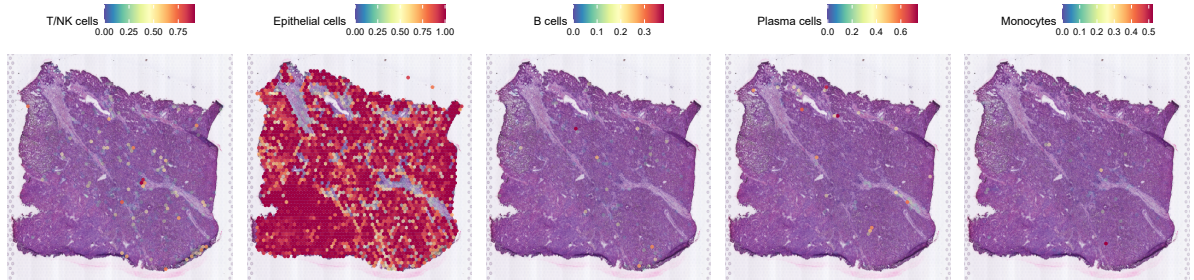

**HCC1T**

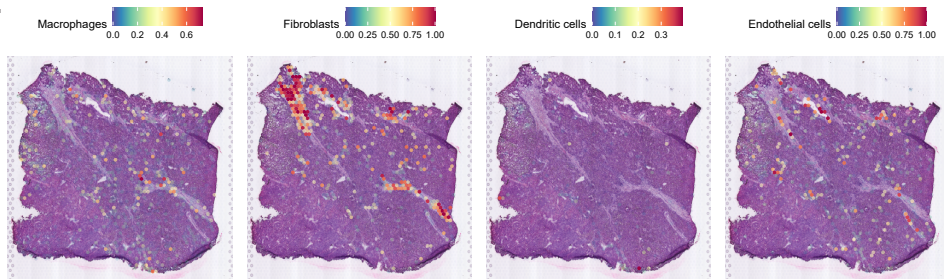

Supplement: Supplementary Figure 2 — Predicted distribution of different cell types in spatial transcriptomic data of MVI-negative Patients. (A, B) Predicted distribution of 9 different cell types in HCC-1L and HCC-1T, respectively. [file Image_2.pdf]

**Scissor0****Scissor-****Scissor+****A**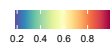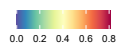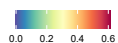**HCC1L**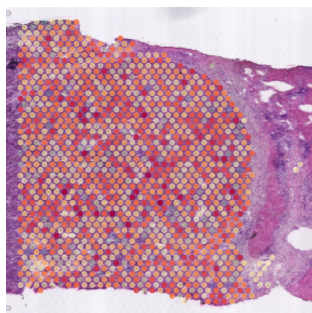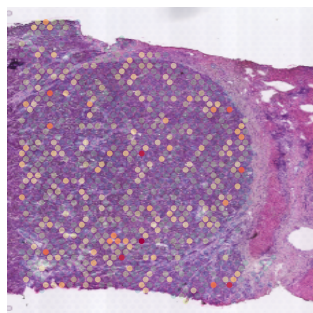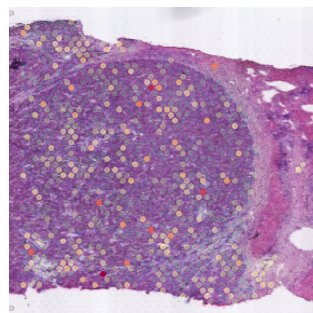**B**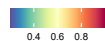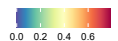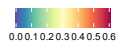**HCC1T**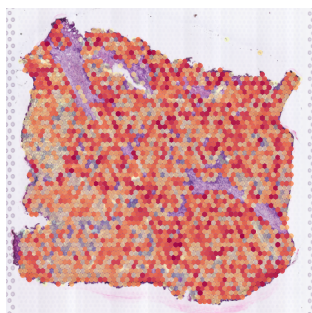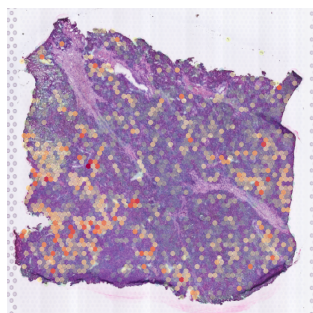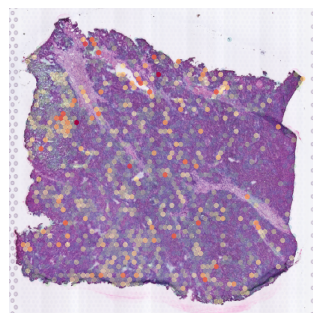**C**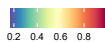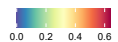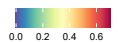**HCC2L**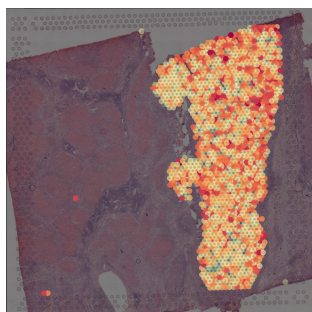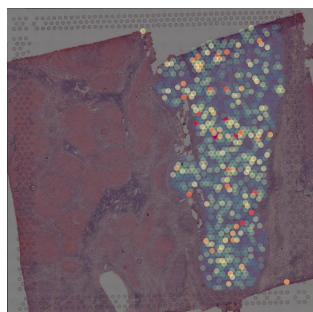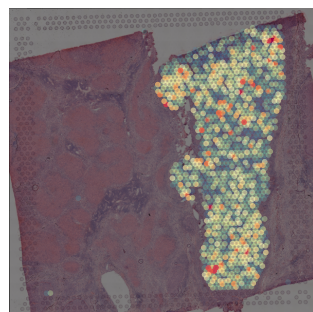**D**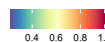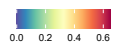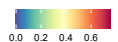**HCC2T**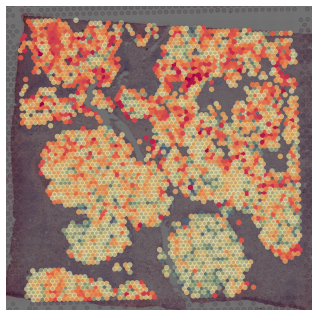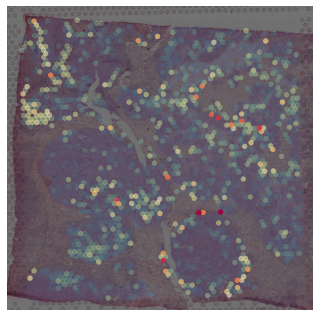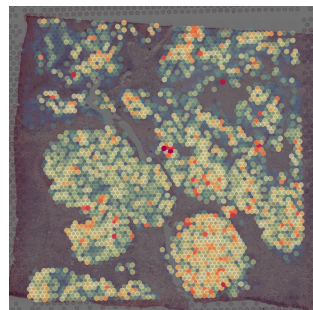**E**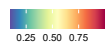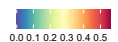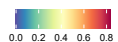**HCC2P**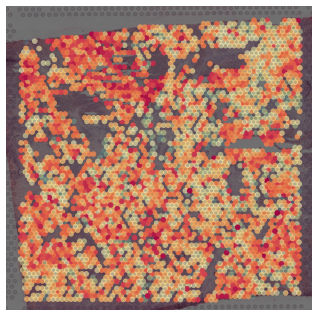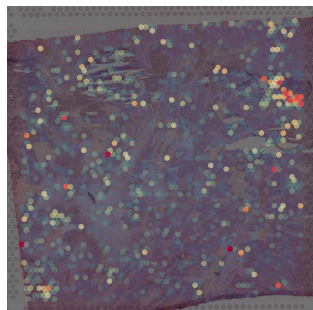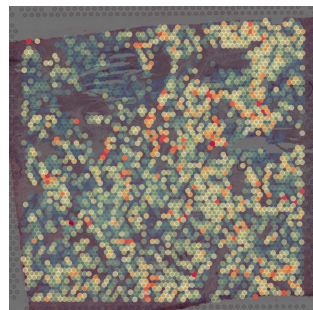

Supplement: Supplementary Figure 4 — Predicted distribution of prognosis-related malignant cells in spatial transcriptomic data. (A–E) Predicted distribution of Scissor0, Scissor-, and Scissor+ malignant cells in HCC-1L, HCC-1T, HCC-2L, HCC-2T, and HCC-2P, respectively. [file Image_4.pdf]
