# Supplementary material for: Multi-transcriptomics analysis of microvascular invasion-related malignant cells and development of a machine learning-based prognostic model in hepatocellular carcinoma
Source: Front Immunol. 2024 Aug 8;15:1436131. doi: 10.3389/fimmu.2024.1436131 (PMC11338809; doi:10.3389/fimmu.2024.1436131)
Supplement: Supplementary Figure 3 — Predicted distribution of different cell types in spatial transcriptomic data of MVI-positive Patients. (A–C) Predicted distribution of 9 different cell types in HCC-2L, HCC-2T, and HCC-2P, respectively. [file Image_3.pdf]

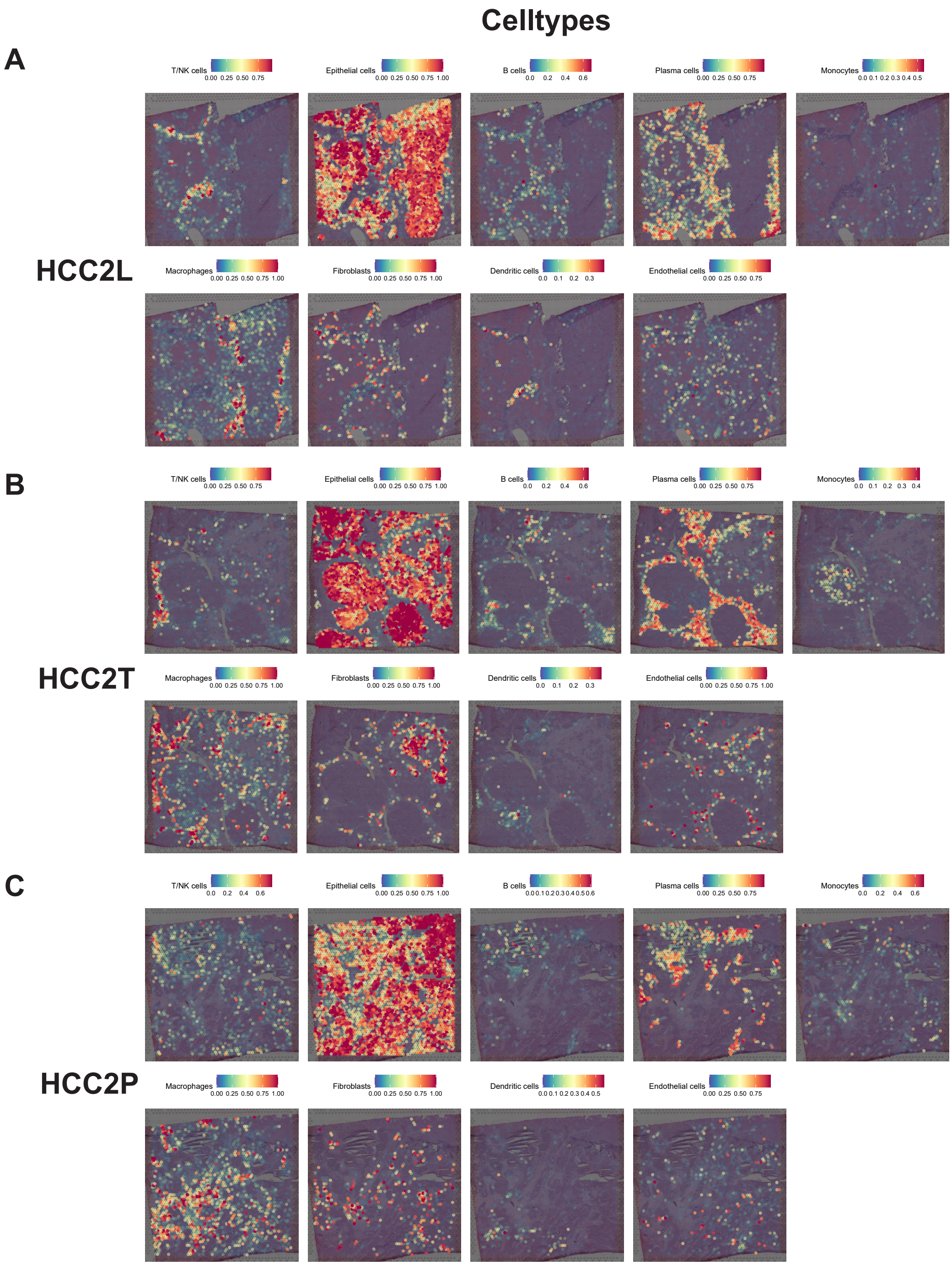
Celltypes
A
HCC2L
T/NK cells
Epithelial cells
B cells
Plasma cells
Monocytes
Macrophages
Fibroblasts
Dendritic cells
Endothelial cells
B
HCC2T
C
HCC2P
